# Evolution of the neural language network

**DOI:** 10.3758/s13423-016-1090-x

**Published:** 2016-07-01

**Authors:** Angela D. Friederici

**Affiliations:** Department of Neuropsychology, Max Planck Institute for Human Cognitive and Brain Sciences, Stephanstraße 1A, 04103 Leipzig, Germany

**Keywords:** Language comprehension, Cognitive neuroscience, Implicit sequence and artificial grammar learning

## Abstract

The evolution of language correlates with distinct changes in the primate brain. The present article compares language-related brain regions and their white matter connectivity in the developing and mature human brain with the respective structures in the nonhuman primate brain. We will see that the functional specificity of the posterior portion of Broca’s area (Brodmann area [BA 44]) and its dorsal fiber connection to the temporal cortex, shown to support the processing of structural hierarchy in humans, makes a crucial neural difference between the species. This neural circuit may thus be fundamental for the human syntactic capacity as the core of language.

The evolution of language has been discussed since Darwin’s *The Descent of Man* in 1871. This discussion mainly focused on nonhuman primates and their cognitive abilities in comparison to human primates. Because of the difficulty of finding evidence for syntax-like abilities in nonhuman primates, a description of the phylogenetic evolution of language remains difficult.

One possibility to approach this issue is comparative: to identify the brain structures that subserve language, and particularly syntactic abilities, in the human brain and to compare these to homologous structures in the nonhuman primate brain. Structural and functional neuroanatomical differences may provide relevant information on the neural basis of the evolution of language. In addition, a comparison between the phylogenesis and ontogenesis of language-relevant brain structures may help to enrich the picture to be drawn on the biological foundation of the language faculty. Here, I will follow this line of examination. I will focus on syntactic structure, because it is a well-defined area that clearly differentiates humans from other animals; however, this is not to suggest that other components of language (phonology, semantics, or pragmatics) are not interesting or important, because these guarantee the communication of meaning.

## Language-related regions

**Fig. 1:**
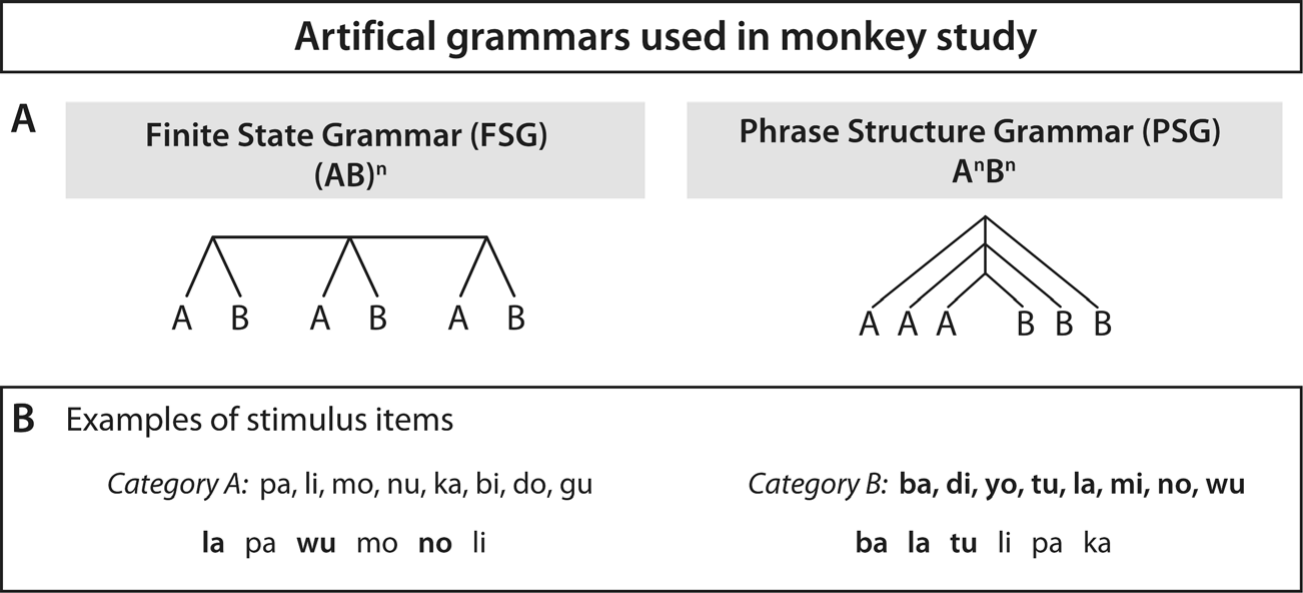
Artificial grammar used in monkey study. Artificial grammar used in the monkey study by Fitch and Hauser (2004). **a** Structure of sequences. **b** Category A syllables and Category B syllables used in the sequences as well as examples of an (AB)^n^ sequence (*left panel*) and an A^n^B^n^ sequence (*right panel*). Category A syllables were produced by a female speaker, Category B syllables by a male speaker. Category membership was thus coded by the pitch of voice. Adapted from “Computational Constraints on Syntactic Processing in a Nonhuman Primate,” by W. T. Fitch and M. D. Hauser, 2004, *Science*, *303*(5656), pp. 377–380. Copyright 2004 by the American Association for the Advancement of Science

Some researchers understand language to cover either all aspects of communication or every step of processing, from the auditory or visual input, through semantic and syntactic processes, to the interpretation and integration of the perceived information into the perceiver’s world knowledge. Others, however, define language as a core faculty responsible for building hierarchical structures, with two interface systems: an external system, serving as a sensorimotor interface, and an internal system, serving as a conceptual-intentional interface (Berwick, Friederici, Chomsky, & Bolhuis, 2013). In the latter view, the core language system is defined as a specific computational mechanism for human language, called “Merge”. This computational mechanism binds two elements (words) syntactically into a novel syntactic unit (phrase). By recursively applying this computation, an unlimited number of sentences can be generated. Thus, Merge is the most basic syntactic computation, which is at the root of any natural grammar sequence and that all human beings appear to posses. Central to the discussion of the evolution of language is not whether sequences can be learned, but more crucially, what *type* of sequences and structures can be learned (Fitch & Friederici, 2012). In this context, a fundamental distinction has often been made between two sequence types with different underlying grammar types: (a) finite state grammars (FSG) following an (AB)^n^ rule and (b) phrase structure grammars (PSG) following an A^n^B^n^ rule (Fitch & Hauser, 2004, see Fig. 1; Hauser, Chomsky, & Fitch, 2002). The crucial differences between these grammars is that in the FSG, the A and B elements stand in an adjacent dependency whereas in the PSG the dependency between A and B elements is nonadjacent.

The schematically drawn relations between A elements and B elements in FSG and PSG in Fig. 1 suggest different underlying structures. However, when thinking about the processes necessary to learn and use these two grammar types, we have to consider not two but at least three possible mechanisms through which these grammatical sequences can be learned. First, adjacent dependencies, as in (AB)^n^ grammars, could be learned by extracting phonological regularities between A and B elements from the auditory input and memorizing these for further use. Second, nonadjacent dependencies between A and B in artificial grammars of the A^n^B^n^ type could in principle be learned through the same mechanism described as the first mechanism (by just memorizing), at least as long as no build-up of a minimal hierarchy is required. Third, the build-up of hierarchies, however, is required in natural grammars, for example, between a determiner (*the*) and a noun (*man*) when building a determiner phrase. This build-up of a phrasal hierarchy is implemented through the computation “Merge” that binds two elements into a minimal hierarchical structure (Chomsky, 1995).

A seminal study (Fitch & Hauser, 2004) compared artificial grammar learning between human and nonhuman primates using FSG and PSG grammar types. Testing cotton-top tamarins and human adults in a behavioral grammar learning study, they found that humans were able to learn both grammar types easily, whereas the monkeys were only able to learn the FSG. These data indicate an essential difference between the species with respect to their grammar-learning abilities. The capacity of other nonhuman primate species to master additional finite-state grammars has also been demonstrated by subsequent studies (Ravignani, Sonnweber, Stobbe, & Fitch, 2013; Sonnweber, Ravignani, & Fitch, 2015). The biological basis for this difference in the different species, however, remained unknown.

With the goal of uncovering the neurobiological basis of processing these grammar types in the human brain, a functional magnetic resonance imaging (fMRI) study in human adults using the same type of grammars was conducted. The data revealed different activation patterns for the processing of (AB)^n^ sequences and A^n^B^n^ sequences (Friederici et al., 2006). Sequences of the PSG activated the posterior portion of Broca’s area (Brodmann area [BA 44]) and the frontal operculum, whereas sequences of the FSG only activated the frontal operculum. Because the frontal operculum is a phylogenetically older brain region (Amunts & Zilles, 2012; Sanides, 1962), the fMRI results in humans may reflect an evolutionary trait.

The specific function of BA 44 and the frontal operculum for the computation Merge has been investigated in a series of fMRI studies (Zaccarella & Friederici, 2015a, b). It was found that although the frontal operculum/anterior insula supports the binding of two elements independent of any phrase structure, BA 44 is recruited only if a hierarchical phrase structure is built. This indicates that BA 44 is the region in the inferior frontal gyrus that particularly subserves syntactic hierarchy building.

Area BA 44, however, is not solely responsible for processing sentential syntax; rather, it is part of a larger frontotemporal network that also includes the posterior temporal cortex (for a review, see Friederici, 2011). The inferior frontal gyrus and the posterior temporal cortex are known to function together during the processing of syntactically complex sentences (den Ouden et al., 2012; Makuuchi & Friederici, 2013). This functional connectivity is made possible by means of dorsally located white matter fiber bundles (i.e., the arcuate fascicle and the superior longitudinal fascicle, which connect the posterior temporal cortex and Broca’s area, in particular BA 44; Anwander, Tittgemeyer, von Cramon, Friederici, & Knösche, 2007; Catani, Jones, & Ffytche, 2005; Friederici et al., 2006).

**Fig. 2:**
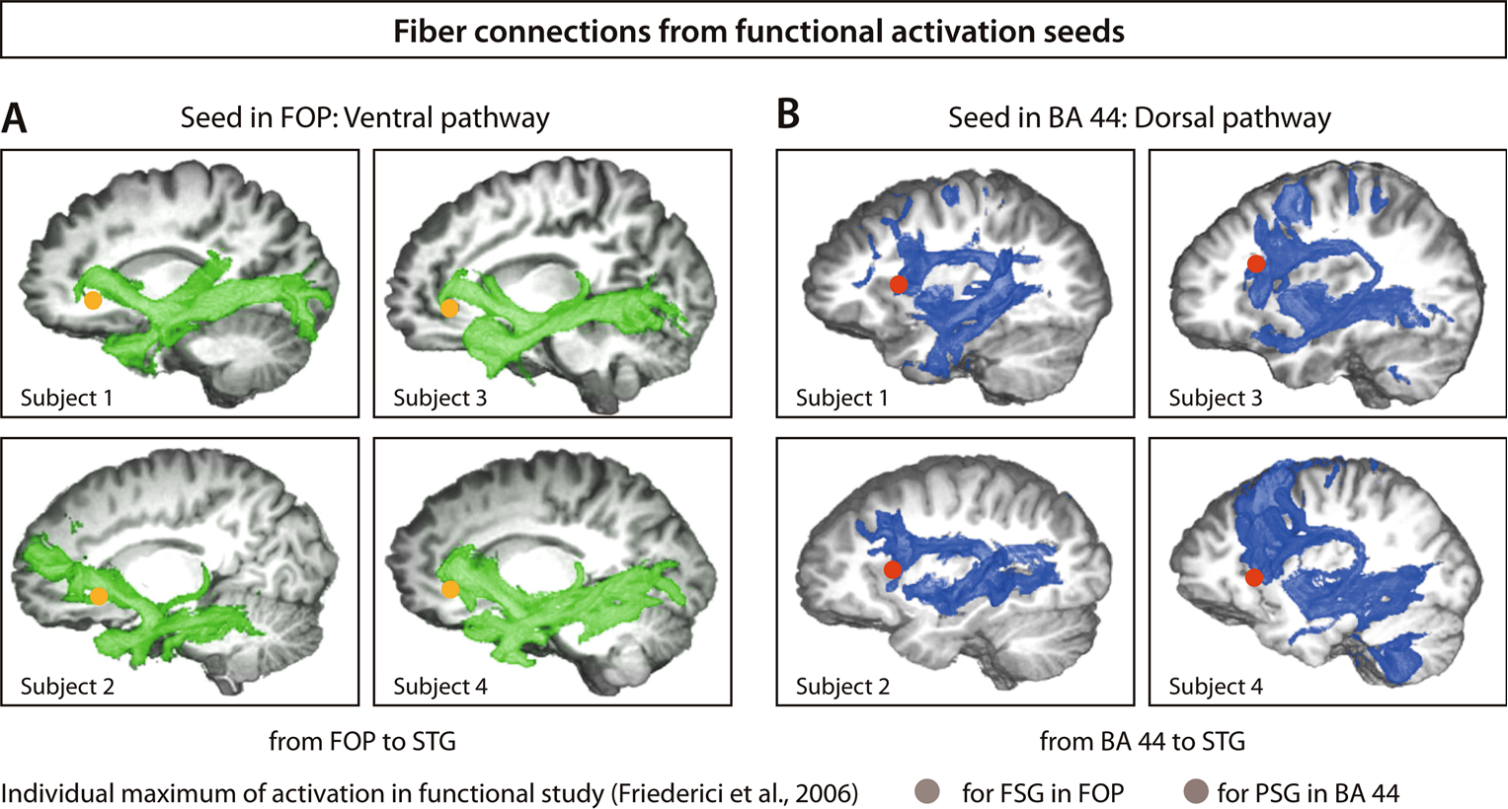
Fiber connections from functional activation seeds. Tractograms for two brain regions: Broca’s area and frontal operculum (FOP). Seed regions were taken from functional on processing (AB)^n^ sequences activating the FOP and A^n^B^n^ sequences activating Broca’s area. Three-dimensional rendering of the distribution of the connectivity values of two start regions with all voxels in the brain volume (*blue*, tractograms from Broca’s area; *green*, tractograms from FOP). **a** Four representative subjects of the group processing a finite-state grammar with their individual activation maxima in the FOP (*orange*) in the critical contrast incorrect versus correct sequences (*p* > .005). For all subjects, connections to the anterior temporal lobe via a ventral pathway were detected. **b** Four representative subjects of the group processing a phrase structure grammar with their individual activation maxima in Broca’s area (*red*) in the critical contrast incorrect versus correct sequences (*p* > .005). For all subjects, the tractography detected connections from Broca’s area to the posterior and middle portion of the superior temporal region via a dorsal pathway. *BA* = Brodmann’s area, *STG* = superior temporal gyrus. Adapted from “The Brain Differentiates Human and Non-Human Grammars: Functional Localization and Structural Connectivity,” by A. D. Friederici, J. Bahlmann, S. Heim, R. I. Schubotz, and A. Anwander, 2006, *Proceedings of the National Academy of Sciences of the USA*, *103*, pp. 2458–2463. Copyright 2006 by The National Academy of Sciences of the USA. (Color figure online)

In addition to these purely functional and purely structural reports on the neural language network, there are studies that combine fMRI and dMRI. One of these studies took the functional activation peaks from artificial grammar processing as seeds for probabilistic tractography, revealing the white matter fiber tracts starting in these seed regions (Friederici et al., 2006). The fMRI experiment applied the FSG and PSG artificial grammar paradigms with rule-based sequences following either adjacent (AB)^n^ or hierarchical nonadjacent A^n^B^n^ dependency rules. The former activated the left frontal operculum, the latter additionally activated the posterior portion of Broca’s area (BA 44; see Fig. 2). The structural data of probabilistic fiber tracking from these two regions revealed two distinct fiber tracts to the temporal cortex: a dorsal pathway connecting BA 44 to the posterior superior temporal gyrus (STG) and middle temporal gyrus (MTG), and a ventral fiber track connecting the frontal operculum to the temporal cortex (see Fig. 2a and b). Because of activation in BA 44 for the processing of nonadjacent, embedded hierarchical dependencies, the dorsal pathway was interpreted to support the processing of complex syntactic structures.

Further evidence for the dorsal fiber tract’s function and relevance for processing syntactically complex sentences comes from patient data and ontogenetic data. The decrease of this fiber tract’s integrity in progressive aphasia is correlated with a decrease in comprehension performance for syntactically complex sentences (S. M. Wilson et al., 2011). The increase of myelination of this fiber tract during development is correlated with an increase in the ability to process syntactically complex sentences (Brauer, Anwander, & Friederici, 2011; Skeide, Brauer, & Friederici, 2015).

**Fig. 3:**
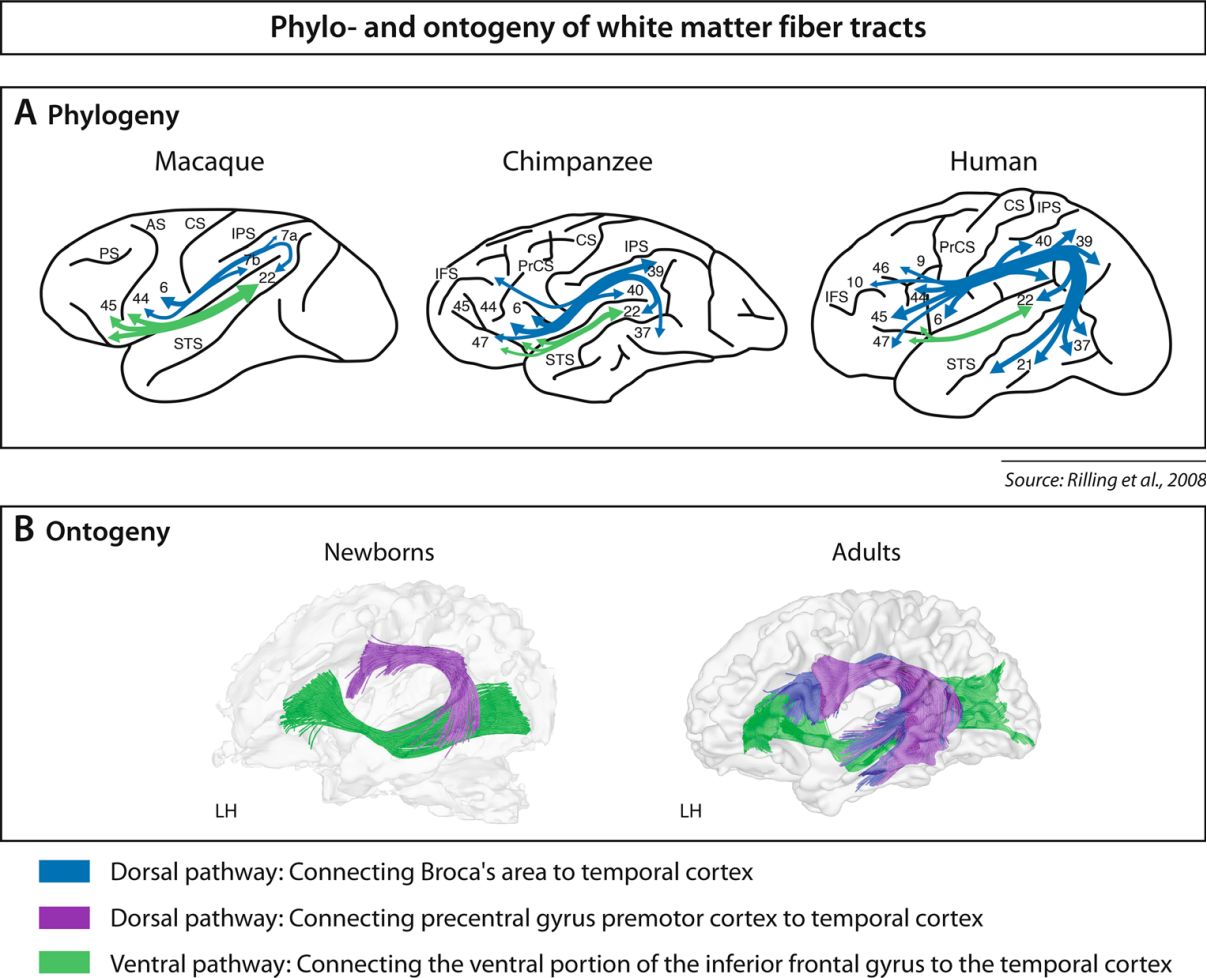
Phylogeny and ontogeny of white matter fiber tracts. **a** Phylogeny. Structural connectivity results. Schematic view. Dorsal fiber tract (*blue*), ventral fiber tract (*green*). Center of gravity of human MTG projections at x ¼ ± 48 are at Montreal Neurological Institute coordinates, x ¼ –48, y ¼ –42, z ¼ –3 and x ¼ 48, y ¼ –36, z ¼ –7. *CS* = central sulcus, *IFS* = inferior frontal sulcus, *IPS* = intraparietal sulcus, *PrCS* = precentral sulcus, *PS* = principal sulcus, *STS* = superior temporal sulcus. Adapted from “The Evolution of the Arcuate Fasciculus Revealed with Comparative DTI,” by J. K. Rilling et al., 2008, *Nature Neuroscience*, *11*(4), pp.426–428. Copyright 2008 by Nature Publishing Group. **b** Ontogeny. Structural connectivity results. Fiber tracking of diffusion tensor imaging data for adults and newborns for speech-relevant regions with seed in Broca’s area and seed in the precentral gyrus/premotor cortex. Two dorsal pathways are present in adults—one connecting the temporal cortex via the arcuate fasciculus and the superior longitudinal fasciculus to the inferior frontal gyrus, that is, Broca’s area (*blue*), and one connecting the temporal cortex to the precentral gyrus, that is, premotor cortex (*purple*). In newborns, only the pathway to the precentral gyrus can be detected. The ventral pathway connecting the ventral inferior frontal gyrus via the extreme capsule to the temporal cortex (*green*) is present in adults and newborns. Adapted from “The Neural Language Networks at Birth,” by D. Perani et al., 2011, *Proceedings of the National Academy of Sciences of the United States of America*, *108*, pp. 16056–16061. Copyright 2011 by the authors and The National Academy of Sciences of the USA

Considering ontogenetic data from birth onward, it is most interesting to see that it is specifically the dorsal fiber tract targeting BA 44 that develops late, and that a second dorsal stream targeting the premotor cortex is already present in the newborn (Perani et al., 2011, see Fig. 3). This latter fiber tract which connects the auditory system to the motor system may be functionally relevant during the infant’s babbling phase, when a coupling of the auditory input and motor output is needed to adjust to the phonology of the language to be learned (Hickok & Poeppel, 2007). The dorsal fiber tract targeting BA 44, however, only becomes relevant later during language development when syntactically complex sentences are to be processed (Skeide et al., 2015). Hence, I conclude that BA 44 as part of Broca’s area and its dorsal connection to the temporal cortex is crucial for syntactic processes in natural languages.

## Cross-species comparisons

Human and nonhuman primates differ in their abilities to process complex sequences. So far, apart from humans, there is no evidence that other species can process and learn hierarchically structured sequences similar to those of natural languages (Beckers, Bolhuis, Okanoya, & Berwick, 2012; Poletiek, Fitz, & Bocanegra, 2016; Yang, 2013). A possible explanation for this behavioral difference may lie in the neural differences between humans and nonhumans with respect to language-related brain structures, in particular.

Language is lateralized to the left hemisphere in the human brain. Neuroanatomically, it has long been reported that the posterior temporal cortex is larger in the left than in the right hemisphere in the human brain (Steinmetz et al., 1989; Watkins et al., 2001; Witelson, 1982), but also in the brain of our closest living relative, the chimpanzee (Gannon, Holloway, Broadfield, & Braun, 1998). It is with respect to Broca’s area that clear neuroanatomical differences among humans and nonhuman primates have been demonstrated: cytoarchitectonic analyses revealed a leftward asymmetry of Broca’s area in humans (Amunts, Schleicher, Ditterich, & Zilles, 2003), but not in the chimpanzee (Schenker et al., 2010). It is interesting to note that during human ontogeny this leftward asymmetry has a different developmental trajectory for the anterior portion of Broca’s area (BA 45) and its posterior portion (BA 44). The left-larger-than-right asymmetry for BA 45, known to serve semantic processes in the adult brain, is present by the age of 5 years, whereas the left-larger-than-right asymmetry for BA 44, known to subserve syntactic processes in the adult brain, only emerges later, by the age of 11 years. These processes, in turn, have different behavioral trajectories in child language development, with semantic processes being established much earlier than syntactic processes, which reach an adult-like performance status much later (Dittmar, Abbot-Smith, Lieven, & Tomasello, 2008; Friederici, 1983). This adult-like behavior co-occurs with the late emergence of adult-like electrophysiological patterns for the processing of complex syntax, only observable after the age of 10 years (see Hahne, Eckstein, & Friederici, 2004). Moreover, it also holds for functional MRI data, revealing a late occurring specificity for syntax in BA 44 (Skeide, Brauer, & Friederici, 2014).

In contrast to humans, the evaluation of the cytoarchitectonically defined Broca’s area in the adult chimpanzee revealed no asymmetry, either in area 45 or in area 44 (Schenker et al., 2010) as homologues to Broca’s area in humans, which is known as a highly language-relevant brain region. Considering the observed cross-species differences concerning the asymmetries, Broca’s area and the development trajectory of its subparts BA 45 and BA 44, it is likely that the observed neurobiological differences between the human and the chimpanzee brain are a crucial parameter for the evolution of language.

When considering white matter brain structure, we observed that, in the human brain, Broca’s area is connected to the posterior superior temporal gyrus and sulcus (STG/STS) via a dorsal white matter pathway (Catani et al., 2005; Rilling et al., 2008). In nonhuman primates, this dorsal pathway is much weaker than in humans (Rilling et al., 2008). A direct comparison revealed differences between humans and nonhuman primates, with macaques and chimpanzees displaying a strong ventral and a weak dorsal pathway whereas humans displayed a strong dorsal pathway and a well-developed ventral pathway (see Fig. 3). The dorsal pathway, which in humans projects into the posterior STG/STS and MTG, was, therefore, discussed as the crucial pathway for the language ability in adult humans (Rilling et al., 2008). Thus, we see a clear evolutionary trajectory across primates with respect to the strength of the dorsal pathway that connects two syntax-relevant brain areas—that is, the posterior portion of Broca’s area (BA 44) and posterior STG/STS. In addition, this pathway also projects to the MTG, known as a brain region to support lexical-semantic processes (Démonet et al., 1992; Vigneau et al., 2006).

Across species, comparisons between the human and nonhuman primate brain thus reveal cytoarchitectonic differences in Broca’s area and connectivity differences between Broca’s area and the temporal cortex. First, cytoarchitectonic analyses demonstrate a leftward asymmetry of Broca’s area in humans, but no such asymmetry in nonhuman primates. Second, the connectivity between Broca’s area and the superior temporal cortex is stronger in the human compared to the nonhuman primate brain. Because Broca’s area and the posterior temporal cortex are the areas that support language processing in the human brain, and because the dorsal fiber tract connecting BA 44 to the STG/STS has been shown to be crucial for processing syntactically complex sentences, these structures present themselves as a possible crucial neurobiological difference for the evolution of language.

But how about the functional similarities and dissimilarities between the species for the language-related brain regions? There are a few studies that provide initial information on this question. A functional difference between human and nonhuman primates was reported for the posterior temporal cortex: Although monkeys activate the STG when listening to monkey vocalizations, human activate both the STG and the STS when listening to human vocalizations (Joly et al., 2012). This is worth noting because the STS and Broca’s area are especially responsive to intelligible human speech.

Comparing the brain basis of artificial grammar processing in human and nonhuman primates, it was found that monkeys (macaques) activate the ventral frontal opercular cortex bilaterally when processing a simple forward-branching grammar (B. Wilson et al., 2015). Humans also show activation in the frontal opercular cortex, but do not recruit Broca’s area (BA 44/45) for this simple forward-branching grammar. The data for the humans is in line with the findings of Friederici et al. (2006), revealing activation in the frontal operculum in response to violations in a simple finite-state grammar sequence.

Another study compared humans and macaques in fMRI experiments on sequence processing and found activation in a number of prefrontal and parietal regions, including the anterior insula for both species (Wang, Uhrig, Jarraya, & Dehaene, 2015). In contrast to macaques, humans demonstrated additional activation in the Broca’s area and superior temporal regions. The involvement of Broca’s area for humans in this study, compared to the previous studies in humans, may be because this study required detection of violations in sequences of tones with respect to their overall numerical and sequential patterns rather than violations of local dependencies in sequences of syllables. The authors take their cross-species data to argue that the frontotemporal circuit observed in humans may have evolved recently and may endow humans with the unique ability of language.

Recent studies suggest that monkeys are able to learn nonhierarchical rule-based sequences (B. Wilson et al., 2015; B. Wilson et al., 2013). This ability has been interpreted as a phylogenetic precursor of processing hierarchical sequences. Similar precursors of processing hierarchical sequences have also been observed in infants (Friederici, Mueller, & Oberecker, 2011; Mueller, Friederici, & Männel, 2012). Both monkeys and human infants are equipped with weak dorsal connections between BA 44 and the temporal cortex. This renders the possibility that both human infants and nonhuman primates show a similar ability in processing rule-based sequences.

Infants have been shown to learn rule-based sequences (Saffran, Aslin, & Newport, 1996) and the rule-based dependency of nonadjacent elements in an auditory sequence (Gómez & Maye, 2005; Mueller et al., 2012) with ease. Such nonadjacent dependencies in auditory sequences in their simplest form are AxB structures in which A and B are constant syllables and the x syllable varies (e.g., as in syllable sequences *le* no *bu*, *le* gu *bu*). The ability to learn such nonadjacent dependencies is taken to be a precursor to learn syntactic dependencies in a natural language. An electrophysiological (EEG) study with 3-month-old infants revealed that violations in AxB sequences were reflected in a mismatch negativity (Mueller et al., 2012). Violations were either acoustic (pitch violation) or rule based (violation of a syllable sequence, AxC instead of AxB). Interestingly, the event-related brain potential (ERP) response in the acoustic pitch-related condition was predictive for the rule-based syllable condition, suggesting a correlation between the ability to detect acoustic violations and the ability to detect violation in rule-based syllable sequences.

To see to what extent human infants and nonhuman primates show similar sequence processing abilities, a recent EEG study with macaques (Milne et al., 2016) used the very same stimulus material as those used by Mueller et al. (2012) with human infants and adults. The ERP results of the monkeys revealed an ERP pattern similar to those of human infants, but different to those of human adults. These data provide support for the view that monkeys’ processing abilities for rule-based sequences are comparable to those of human infants. This goes together with a structurally comparable maturational and evolutionary status of the dorsal fiber tract connecting BA 44 and posterior temporal cortex.

## Conclusion

The data discussed lead to the tentative conclusion that the neural circuit, consisting of BA 44 and the posterior superior temporal cortex connected via a dorsally located fiber tract, may be seen as a crucial evolutionary advancement toward the unique human faculty of language.
